# Enhancing the quality of cognitive behavioral therapy in community mental health through artificial intelligence generated fidelity feedback (Project AFFECT): a study protocol

**DOI:** 10.1186/s12913-022-08519-9

**Published:** 2022-09-20

**Authors:** Torrey A. Creed, Leah Salama, Roisin Slevin, Michael Tanana, Zac Imel, Shrikanth Narayanan, David C. Atkins

**Affiliations:** 1 Perelman School of Medicine, University of Pennsylvania, Philadelphia, USA; 2 Lyssn.io, Inc, Seattle, USA; 3 Department of Educational Psychology, University of Utah, Salt Lake City, USA; 4 Viterbi School of Engineering, University of Southern California, Los Angeles, USA; 5 Department of Psychiatry and Behavioral Sciences, University of Washington School of Medicine, Seattle, USA

**Keywords:** Artificial intelligence, Cognitive behavioral therapy, Fidelity, Competence, Community mental health, Training, Supervision, User-centered design, Technology, Implementation science

## Abstract

**Background:**

Each year, millions of Americans receive evidence-based psychotherapies (EBPs) like cognitive behavioral therapy (CBT) for the treatment of mental and behavioral health problems. Yet, at present, there is no scalable method for evaluating the quality of psychotherapy services, leaving EBP quality and effectiveness largely unmeasured and unknown. Project AFFECT will develop and evaluate an AI-based software system to automatically estimate CBT fidelity from a recording of a CBT session. Project AFFECT is an NIMH-funded research partnership between the Penn Collaborative for CBT and Implementation Science and Lyssn.io, Inc. (“Lyssn”) a start-up developing AI-based technologies that are objective, scalable, and cost efficient, to support training, supervision, and quality assurance of EBPs. Lyssn provides HIPAA-compliant, cloud-based software for secure recording, sharing, and reviewing of therapy sessions, which includes AI-generated metrics for CBT. The proposed tool will build from and be integrated into this core platform.

**Methods:**

Phase I will work from an existing software prototype to develop a LyssnCBT user interface geared to the needs of community mental health (CMH) agencies. Core activities include a user-centered design focus group and interviews with community mental health therapists, supervisors, and administrators to inform the design and development of LyssnCBT. LyssnCBT will be evaluated for usability and implementation readiness in a final stage of Phase I. Phase II will conduct a stepped-wedge, hybrid implementation-effectiveness randomized trial (*N* = 1,875 clients) to evaluate the effectiveness of LyssnCBT to improve therapist CBT skills and client outcomes and reduce client drop-out. Analyses will also examine the hypothesized mechanism of action underlying LyssnCBT.

**Discussion:**

Successful execution will provide automated, scalable CBT fidelity feedback for the first time ever, supporting high-quality training, supervision, and quality assurance, and providing a core technology foundation that could support the quality delivery of a range of EBPs in the future.

**Trial registration:**

ClinicalTrials.gov; NCT05340738; approved 4/21/2022.

## Background

There is a mental health crisis in the United States. One out of five adults will receive a mental health diagnosis in their lifetime [1], and major depression is currently the single biggest contributor to disability globally [2]. Over the past several decades, scientists have demonstrated the efficacy and cost-effectiveness [3, 4] of treatments for mental health disorders, including psychosocial interventions such as Cognitive Behavioral Therapy (CBT) [5, 6]. Despite the billions of dollars spent to disseminate evidence-based psychotherapies (EBPs) like CBT into clinical settings [5–9], access to these effective treatments remains severely limited. Training, policy mandates, and value-based incentives have not translated to broad access to high-quality EBP care in the community [10–12]. A specific barrier to effective implementation and sustainment of psychosocial interventions is the ability to measure therapist fidelity. Proctor et al. concluded that “The foremost challenge [to disseminating EBPs] may be *measuring implementation fidelity quickly and efficiently*” ([13], p. 70; italics added). To effectively implement psychosocial interventions in community settings and capitalize on the significant investment that health systems have made in EBPs, technology is needed to scale up fidelity assessment “quickly and efficiently”.

While measurement-based care has demonstrated differences in the effectiveness of providers [14], the existing technology for evaluating therapist EBP fidelity and quality does not scale up to real-world use. Specifically, the research-based, gold-standard for assessing fidelity is behavioral coding: A session is recorded, and then this “raw data” is rated by trained human coders. Research on training and quality assurance indicates that using objective, performance-based feedback like behavioral coding can enhance and sustain therapist skills [15], and ultimately client access to EBPs such as CBT [16, 17]; without performance-based feedback or quality monitoring, the return on investment of costly implementation efforts is often lost [15, 18]. However, this process is time consuming, expensive, and at times, error prone: It is a non-starter in the vast majority of community practice settings. Accordingly, mental health services researchers utilize a variety of alternative measures, including patterns of utilization (e.g., continuity of care, the number of sessions in a specified time period after diagnosis), therapist self-reports of adherence, client-rated measures of satisfaction, or measures of clinical outcomes [19, 20]. However, these are proxies of intervention quality, distal to the content of the clinical encounter, and/or subject to self-observation bias. They are all problematic indicators of fidelity and quality. Behavioral coding provides a methodology for measuring EBP fidelity, but it is impractical at scale, forcing reliance on feasible but circumspect metrics [13, 15].

Advances in machine learning (ML) and artificial intelligence (AI) have transformed computers’ abilities to create, understand, and respond to natural language. There have been major advances in basic processes (e.g., natural language understanding), as well as consumer-facing technologies (e.g., Alexa, Siri). In addition, cloud-based computing means that any internet-connected device can access server-based computing power that can scale on-demand. *Lyssn.io*, Inc, (or Lyssn, pronounced ‘listen’) is a technology start-up deploying an array of such AI technologies to support training, supervision, and quality assurance of EBPs.

Lyssn has an established cloud-based platform which includes: a) user management and organization of sessions, clinicians, and supervisors, b) recording, playback, and annotation of audio or video data from therapy sessions; c) speech-to-text transcription, d) AI-generated fidelity and quality metrics; and e) data summaries and visualizations for feedback. Therapists and supervisors access Lyssn via a web-browser, and a therapist’s caseload of patients is shown in a dashboard. Via the web-based dashboard, therapists can record in-person or telehealth sessions or upload sessions recorded elsewhere. The session review interface enables time-linked comments directly in the video (or audio) playback, which facilitates efficient use of traditional supervision. The platform allows therapists and their supervisors to discuss a session asynchronously and immediately queue up a portion of the session to review. In addition, each session is automatically transcribed via Lyssn’s in-house, state-of-the-art speech recognition algorithms, trained on over 4,000 sessions. Lyssn’s algorithms automatically identify separate speakers and their role (i.e., client vs therapist), and the transcript is searchable and linked to the recording to support efficient review and supervision [21–23]. This supervision platform serves as a base for the AI-generated psychotherapy quality metrics.

Study team members led the foundational research that established that ML-based evaluation of psychotherapy quality is possible [24]. The Lyssn platform incorporates algorithms that automatically identify Motivational Interviewing (MI) fidelity codes from session recordings. These algorithms utilize speech and language features to identify both session-level (e.g., how empathic was the therapist in this session?) and per-utterance (e.g., open questions, affirmations, confrontations within talk-turns) MI fidelity codes. The study team has published numerous papers on machine learning applied to MI and psychotherapy common factors (e.g., therapeutic alliance, facilitative interpersonal skills; [25–48]). The proposed work will extend the AI aspects of the Lyssn platform to CBT and further develop the Lyssn user-interface to support community mental health needs and workflows.

The Lyssn MI psychotherapy quality metrics have recently been extended to CBT, building on a 15-year partnership between the Penn Collaborative and Philadelphia’s Department of Behavioral Health and Intellectual disAbillity Services (DBHIDS) to implement CBT in Philadelphia’s community mental health (CMH) system, known as the Beck Community Initiative (BCI; [49]). After an initial implementation readiness phase, intensive workshops teach CBT theory and strategies, followed by weekly group case consultation. In the 6-month consultation phase, therapists’ CBT competence is rated from recorded therapy sessions using the Cognitive Therapy Rating Scale (CTRS; [50–52]). BCI training significantly improved CBT quality [49]. Prior to training, only 2% of therapists demonstrated CBT competence, while the majority of therapists (79.6%) demonstrated competence by the end of training [53]. While mean final competence scores (*M* = 41.2) were above the criterion threshold by the certification point, there remains an opportunity to improve skills among those who have completed training, as well as those who continue to participate in BCI training. Considerable research [15] also shows that training effects wear off over time without additional support, such as performance-based feedback, but this requires ongoing and extensive efforts (i.e., CTRS coding) that are not income-generating or reimbursed by payers. Clearly, human-based fidelity coding presents a substantial challenge in the training protocol. BCI CTRS coding to date is the equivalent of a doctoral-level rater working full-time for almost 4 years, or approximately $100-$125 per session rated—more than the cost (reimbursement) of the session itself. It is expensive, a rate limiting factor for scaling up training, and does not provide for sustainability, as ongoing CTRS coding ends at 6 months post-workshop. Using technology instead of humans for feedback on CBT competence would promote scale, efficiency, sustainability, and more effective allocation of limited human training resources.

**Fig. 1:**
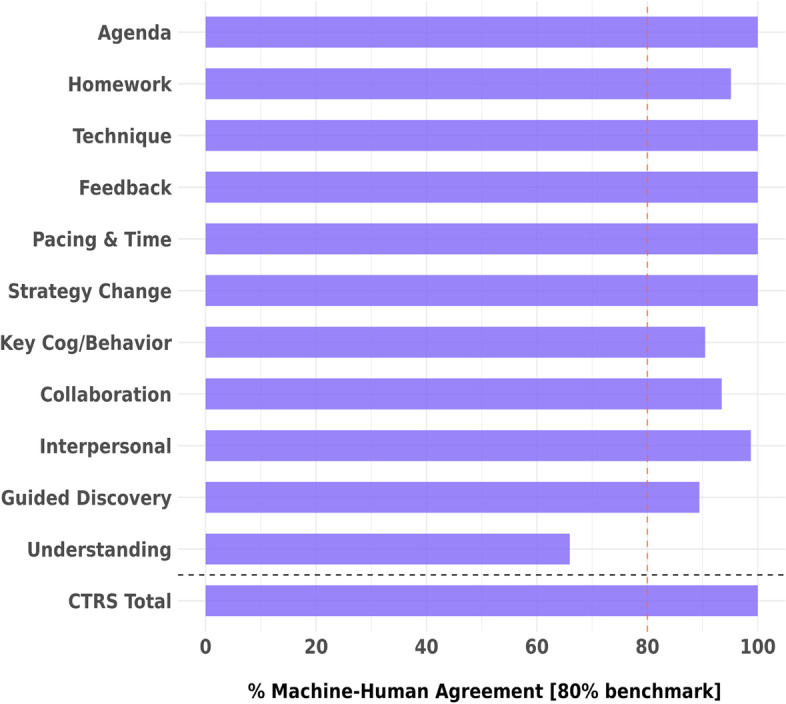
Percentage agreement between AI-generated and human-generated CTRS codes

Supported by R56 MH118550, the study team demonstrated initial feasibility of using ML models to rate CBT fidelity (i.e., CTRS codes; [50]) from linguistic features using a subset of sessions from previous BCI trainings [54, 55]. In 2020, Lyssn established a data use agreement with the University of Pennsylvania for the updated corpus of recordings (*n* = 2,494) and related CTRS ratings. Using transformer-based, deep neural networks [56], Lyssn developed AI-generated models for each of the 11 CTRS codes using all 2,494 sessions (within a cross-validation framework of test and training partitions; [57, 58]). The goal is that AI-generated metrics are indistinguishable from human-generated metrics, with a benchmark of 80% of human reliability (e.g., if human reliability is 0.80 and AI predicted scores correlate with human scores at 0.75, then AI predictions are 0.75 / 0.80 or 94% of human reliability). All results are based on a 30% test set of sessions that is totally distinct from the training set where models were originally developed and fit. Results shown in Fig. 1 demonstrate very strong signal and prediction. In all but one instance (CTRS code Understanding), AI-generated predictions cross the 80% of human reliability threshold. It is worth noting that in almost all clinical research using the CTRS, reliability estimates focus exclusively on the CTRS total score [49, 59]. Using AI-generated metrics, the tools achieve 100% of human reliability on the total score and also demonstrate highly accurate individual item reliability. This published research and large-scale analyses of the Penn Collaborative’s CTRS data demonstrate feasibility for the development of a LyssnCBT tool for automated fidelity feedback. Moreover, Lyssn has a standing in-house coding team that continuously provides new validation and calibration data to assess the ongoing performance of the productive AI algorithms.

### Objectives and aims

The primary objectives of Project AFFECT (AI-Based Fidelity Feedback to Enhance CBT) are to 1) refine a LyssnCBT user interface geared to CMH clinical, supervision, and administrative workflows and needs and evaluate it for usability and implementation readiness and 2) prospectively evaluate both service and implementation outcomes using LyssnCBT for supervision and quality assurance. LyssnCBT will massively scale up evaluation and feedback capacity to support high-quality CBT in routine care settings across the US. The end goal is more therapists across the country providing higher quality CBT to the millions of Americans suffering from mental health challenges.

Project AFFECT has two phases. In Phase 1 the objective is to use an existing prototype to develop LyssnCBT for CMH settings and evaluate its usability. This will include: understanding community stakeholder needs to inform software design and functionality (Aim 1), and evaluating usability and implementation readiness of LyssnCBT with CMH therapists and supervisors in a standardized roleplay design (Aim 2). Iterative software development and preliminary system validation will ensure readiness to advance to Phase 2 testing. The objective of Phase 2 is to evaluate LyssnCBT in real-world, CMH settings. A hybrid type 2 implementation-effectiveness, stepped-wedge randomized study of LyssnCBT will evaluate improvement in CBT skill use and client outcomes with 50 therapists and 1,875 clients across 5 CMH clinics (Aim 1). In addition, the hypothesized mechanism by which LyssnCBT affects clinical outcomes will be assessed (Aim 2).

## Methods/design

### Study setting

DBHIDS is a $1 billion per year healthcare system with over 300 agencies that provide behavioral health services to the city’s 470,000 Medicaid recipients, plus thousands of uninsured and underinsured individuals [51]. To qualify for services, individuals must live in Philadelphia and earn no more than 138% of the federal poverty index. The DBHIDS client population is racially and ethnically diverse (e.g., 50.1% Black / African-American, 24.8% Latino / Hispanic, 21.4% White / Caucasian) and over 54% of clients are women. Clinics treat individuals with a broad range of mental health and substance use problems: Depression (30.6%), Substance Use / Dependence (28.9%), Bipolar / Other Mood Disorders (26.7%), Psychotic Disorders (13.3%), and Anxiety Disorders (12.8%; [60]). Additional therapists are enrolled in the BCI each year through its contracts with DBHIDS.

### Participants and procedures

For Phase 1, Project AFFECT will recruit therapists, supervisors, and administrators (*n* = 25) from within the DBHIDS network. Inclusion criteria will include being currently employed at an adult outpatient CMH program that has received CBT training and implementation support from the BCI and being able to engage in study processes in English. For Phase 2, the Project AFFECT study team will identify 5 adult outpatient CMH programs from among the BCI partner programs who agree to integrate LyssnCBT into their routine procedures. Across the 5 agencies, therapists (*n* = 50) and their supervisors will be recruited for participation. Clients from the caseloads of participating therapists will be recruited to participate, with a goal of 5 consenting clients per therapist. Median treatment length at DBHIDS programs is approximately 10 weekly sessions. Across 18 months of planned data collection (~ 75 weeks), a minimum of 1,875 clients for 50 therapists are expected (i.e., 50 therapists × 5 sessions per week × 75 weeks = 18,750 sessions, with an average of 10 sessions per client).

### Phase 1 research design and methods (1 year)

The goal of Phase 1 is to refine a fully functional prototype of LyssnCBT designed for CMH use and workflows. It will be integrated within the Lyssn cloud-based, software platform and make use of previously developed AI models for CBT fidelity. The prototype will be evaluated for usability and implementation readiness, and the Phase 1 milestones will establish readiness for a randomized evaluation of LyssnCBT in Phase 2.

#### Aim 1: Community Mental Health (CMH) user-centered design and software development

The LyssnCBT user-interface (UI) for CMH settings and workflows will be refined using an iterative, user-centered design (UCD) process so that the front-end UI of the system is maximally useful (and implementable) to a variety of end-users. Three groups of stakeholder participants will be recruited from sites previously trained in CBT by the BCI: therapists (*n* = 3), supervisors (*n* = 3) and clinic administrators (*n* = 4). Participants will be compensated $50 for their participation. The focus group and individual interviews will probe: typical client population, clinical and supervision workflows, current information technology (IT) infrastructure, and (administrators only) how quality assurance is currently conducted. A brief demonstration of the Lyssn platform and the existing CBT fidelity prototype will be provided. Participants will be queried about whether and how CTRS-based feedback is currently used within ongoing supervision, additional features of sessions and/or clients that would be useful for the LyssnCBT system to capture and report back, and perceived motivators and barriers to adoption of the LyssnCBT system.

Based on the input from the UCD design sessions, the research team will refine the design of the LyssnCBT software. It will build from Lyssn’s HIPAA-compliant cloud system, and while final features and functions will be shaped by the design sessions, the LyssnCBT UI is anticipated to include interactive summaries of CBT fidelity scores (individual items, plus total), allowing summarization of ranges of sessions, clients, and therapists along with drill down to individual sessions for review and supervision.

#### Aim 2: usability and implementation readiness of LyssnCBT with standardized patients

After the LyssnCBT software is adjusted to reflect the UCD feedback, usability and implementation readiness will be assessed using standardized patient (SP) methodology. Ten CMH therapists and five supervisors will be recruited to participate in an individual, 60–90 min session, including an SP “therapy” session and semi-structured interview. (Note: Supervisors will not record SP sessions and interview questions will be framed around supervision processes, but otherwise will be largely similar to therapist sessions.) There will be a brief introduction to the Lyssn recording platform, and then each therapist will use it to record a 15-min session with the SP (played by a study team member), treating it as if it were a regular therapy session at their clinic. The recorded therapy session will be processed by LyssnCBT. The therapist participants will then be guided through the LyssnCBT interface, which will display the SP session just recorded along with other roleplayed sessions pre-recorded by the research team. During the semi-structured interview, participants will be solicited for feedback on UI elements, including visualizations of CBT measures, data fields, and navigation controls. Participants will then be asked how they would imagine using LyssnCBT to complete critical actions, like assessing their performance during a clinical session or reviewing a session during supervision. At the end of the sessions, therapist and supervisor participants will complete brief (4-item) implementation measures of acceptability (Acceptability of Intervention Measure; AIM; [61]), appropriateness (Intervention Appropriateness Measure; IAM; [61]), and feasibility (Feasibility of Intervention Measure; FIM; [61]), as well as usability (System Usability Scale; SUS; [62]) with respect to the LyssnCBT prototype. The research team will review and refine the LyssnCBT platform based on the feedback gathered in this stage.

### Phase 2 research design and methods (3 years)

The primary research activity of Phase 2 is a type 2 hybrid implementation-effectiveness, randomized study comparing LyssnCBT for clinical and supervision services to services as usual (SAU), where the primary outcomes include therapist CBT skill and client outcomes (symptom improvement and drop-out). Working closely with DBHIDS leadership, 5 programs will be recruited from among the Penn Collaborative / BCI CMH partner organizations, targeting adult outpatient mental health clinics with 8 or more staff therapists. Agency participation will include the integration of LyssnCBT on a program level to facilitate the integration of LyssnCBT into standard workflow practices including requesting consent to record and participate from clients during the standard intake process, and integration into supervision practices. Therapists (*n* = 50) and their supervisors will be recruited, where each therapist will have a caseload of about 5 clients participating in the study at any given time. (Note: Participating therapists may use LyssnCBT with as many of their clients as they wish, but they will be asked for a minimum of 5 clients consenting to participate in study data collection at any given time.) Median treatment length at DBHIDS clinics is approximately 10 weekly sessions. Across 18 months of planned data collection (~ 75 weeks), recruitment is expected to yield a minimum of 1,875 clients (i.e., 50 therapists × 5 sessions per week × 75 weeks = 18,750 sessions, with an average of 10 sessions per client).

**Fig. 2:**
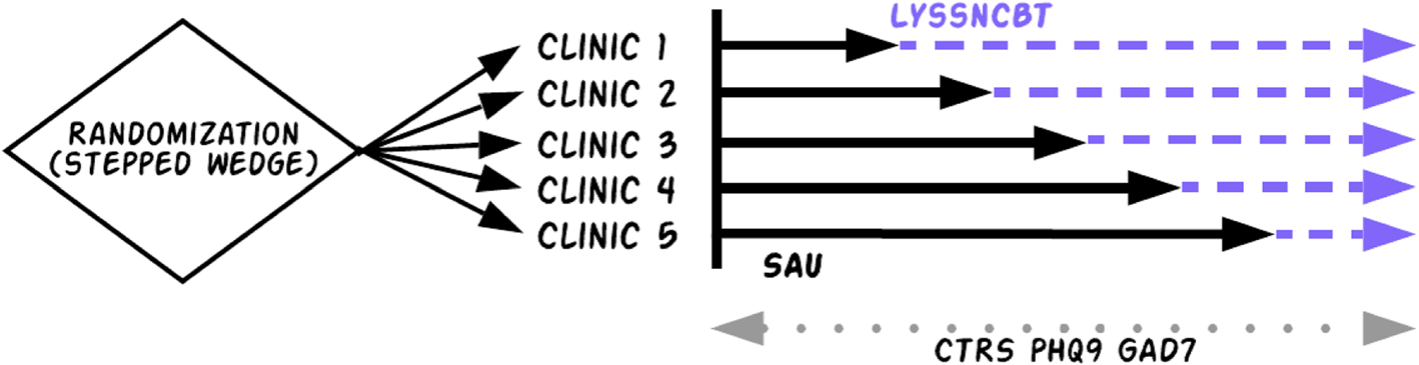
Phase 2 study design overview

LyssnCBT will be compared to SAU using a stepped-wedge design in which each clinic will have SAU and LyssnCBT phases. Stepped wedge designs allow the intervention (here, LyssnCBT) to eventually roll out to all clinics and therapists and also has greater power than a parallel cluster randomized trial [63]. As shown in Fig. 2, all 5 clinics will start with SAU (black solid lines), and clinics will be randomized to begin LyssnCBT sequentially over time (dashed purple) using simple randomization. The names of the five participating agencies will each be enclosed in individual sealed envelopes, and every two months, a study team member will select one envelope with the name of the agency to begin LyssnCBT. At the start of the trial when all clinics are in the SAU phase, all clinics will begin using a modified version of the Lyssn platform for recording sessions that provides access to the recording and session sharing functionality, but no other features (e.g., speech-to-text transcription, annotation tools, any AI-generated metrics). When a clinic is randomly selected to use LyssnCBT, there will be an onboarding and training session to cover the software and clinical / supervision protocols. Participants may withdraw or take away permission to use and disclose their information at any time by sending written notice to the investigator for the study. If they withdraw their permission, they will not be able to stay in the study.

#### Assessments / outcomes

There are two classes of primary outcomes: a) therapist CBT fidelity, and b) client outcomes. CBT fidelity will be assessed by AI-generated CTRS scores for every recorded therapy session, which will be recorded via the Lyssn platform during both SAU and LyssnCBT phases of the study (approximately 18,750 sessions in total). Client outcomes will be evaluated using the Patient Health Questionnaire-9 (PHQ-9) and Generalized Anxiety Disorder-7 (GAD-7) at each session. The PHQ-9 is a brief, widely used depression inventory with 9 total items, and similarly, the GAD-7 is a brief, widely used anxiety inventory with 7 total items [64–66]. The PHQ-9 and GAD-7 are reliable and valid and can be completed in 2–3 min. These client self-report measures will be collected via a web-based survey tool that supports text and email notification via URL to complete assessments and integrates with the Lyssn platform. Before a participating therapist begins a new session using the Lyssn platform, an email (or text) notification will be sent to the client to complete the PHQ-9 and GAD-7. Data will also be collected on client drop-out / premature termination via a brief monthly survey sent out to participating therapists. Finally, after three months of engagement with the LyssnCBT tools, each participating therapist and supervisor will complete the battery of implementation measures, including the SUS, AIM, IAM, and FIM, as well as have an opportunity to provide more general feedback on the LyssnCBT system. All assessments are summarized in Table 1. Therapists and supervisors will receive a gift card worth $30 for attending a Lyssn onboarding session and $20 for completing implementation measures. In addition, therapists will be compensated $15 per participating client. Clients will be compensated with one $10 gift card for allowing the research team access to the data from their weekly symptom measures and session recordings.

**Table 1:** Summary of Phase 2 data collection

Participant	Construct	Measures	Frequency and timeline
*Client*	Depression, Anxiety	PHQ-9, GAD-7	Before each session
	Drop-out from treatment	Survey	Once a month
*Therapist*	CBT fidelity	CTRS	After each session
	Implementation outcomes	AIM, IAM, FIM, SUS	Once, 3 months after start of LyssnCBT phase
*Supervisor*	Implementation outcomes	AIM, IAM, FIM, SUS	Once, 3 months after start of LyssnCBT phase

*PHQ-9* Patient Health Questionnaire-9, *GAD-7* Generalized Anxiety Disorder-7, *CTRS* Cognitive Therapy Rating Scale, *AIM* Acceptability of Intervention Measure, *IAM* Intervention Appropriateness Measure, *FIM* Feasibility of Innovation Measure, *SUS* System Usability Scale

#### Data analysis plan

CBT fidelity, client symptom measures (PHQ-9, GAD-7), and client drop-out will be analyzed with mixed-effects models (also called hierarchical or multilevel models; [67–70]). Mixed models are very flexible with respect to nested and imbalanced data, where the current data will contain repeated measures within clients and within therapists with varying numbers of sessions and clients. Within each individual clinic, a stepped wedge design is similar to an interrupted time series, with pre-intervention (i.e., SAU) and post-intervention (i.e., LyssnCBT) phases. To model the intervention effect, separate slopes will be examined for time by phase to capture differential changes in outcomes across the two phases. *Condition* (LyssnCBT vs SAU) will be dummy-coded, and clinics will also be included in the model as dummy-coded control variables. Analyses across the outcomes will be very similar, with the exception that client drop-out is a binary outcome, whereas a logistic mixed effects model will be used instead. The primary focus in all analyses is the main effect of *Condition* and its interaction with *Time* (during the phase since LyssnCBT started). Finally, sensitivity analyses will examine missing data. Missing data could be a function of a therapist not recording a session, or of a client not completing self-report measures. The research team will review weekly reports of anticipated recordings and assessments to prevent missing data whenever possible. No significant challenges with missing data are anticipated, though calculations have assumed 20% attrition in power analyses (see below). Mixed-effects models provide unbiased estimates in the presence of missing data as long as missing data can be assumed Missing at Random (MAR; [70]). If missing data is greater than 20% or there are other concerns about the MAR assumption, a pattern-mixture approach will be used to (potentially) non-ignorable missing data [71].

##### Conceptual model and evaluation of the proposed mechanism of action

According to the deliberate practice model, both repetition and specific, performance-based feedback are crucial to improving provider skill [72–75]. LyssnCBT is designed to enhance exposure to repeated practice opportunities (i.e., CBT sessions) with exactly the type of specific, performance-based feedback emphasized in the deliberate practice model. In support of this, high-quality implementation efforts that are inclusive of practice with feedback increase CBT competence [49]. The skills training involved in CBT (encompassing both cognitive interventions like generating alternative explanations and cognitive coping skills, and behavioral interventions, like behavioral activation and coping skills) act as mediators in reducing distress and impairment among individuals with mental health problems. Adopting principles of experimental therapeutics outlined in the NIMH strategic plan [76] and applying them to the conceptual frameworks guiding LyssnCBT, the following mechanism of action will be assessed: LyssnCBT will provide performance-based feedback on CBT fidelity, which should improve therapist CBT skills which in turn should improve client outcomes. Importantly, most process or mechanism research has been limited to 100–200 sessions due to human-based observational coding [77]. In the present study, the proposed mediation model above will be assessed using more than 18,000 sessions.

The hypothesized mediation model will be tested using mixed models [78]. Specifically, analyses will test the total effect (or “c” pathway in mediation literature) of LyssnCBT on client outcomes (i.e., PHQ-9, GAD-7, premature drop-out), then the effect of LyssnCBT on CTRS scores (or “a” pathway). Finally, analyses will test the direct effect of LyssnCBT on client outcomes (“c prime”) while controlling for the effect of the CTRS mediator (“b” pathway). The indirect (“a*b”) effect will be tested via bootstrap confidence intervals [79] and is a direct estimate of the hypothesized mechanism. Two additional analyses are also planned. While the above analyses provide the traditional approach to mediation, it is specifically hypothesized that it is *changes* in CBT fidelity (i.e., CTRS scores) due to LyssnCBT which would drive improved client outcomes. To examine this hypothesis, CTRS deviation scores will be created. Mean CTRS scores during SAU will be estimated for each therapist and for each CTRS item (i.e., mean *Agenda* score during SAU phase). These means will be subtracted from each corresponding CTRS score during the LyssnCBT phase (e.g., if mean *Agenda* during SAU is 2.5 and 6 in a new session, the deviation score would be + 3.5). These CTRS deviation scores will provide somewhat more specific information on whether the improvement in CBT fidelity is driving client outcomes. Finally, the Lyssn platform collects user-interaction data, which will be used to examine whether time spent reviewing sessions and interacting with the LyssnCBT UI are predictive of improved client outcomes.

##### Power and sample size

Power and sample size estimation took into account a number of factors: 1) correlation of CBT fidelity and client symptoms within therapists, 2) repeated measures, 3) stepped wedge design, and 4) a range of possible effect-sizes. Intraclass correlation coefficients (ICC; i.e. the correlation in the data due to nesting) were based on recent analyses of more than 400 CBT therapists and associated CBT fidelity ratings (ICC = 0.20; [80]) and published literature on patient symptoms (ICC = 0.10; [14]). Power and sample size focused on the effect of LyssnCBT on client outcomes, where a smaller effect size is expected as compared to therapist CBT skill, which is being directly targeted by LyssnCBT. Sample size was estimated using the formula for mixed model power analysis developed by Raudenbush and colleagues [81] that incorporates therapist ICCs and repeated measures, used in conjunction with a design effect from the specific stepped-wedge design proposed [66]. Because stepped-wedge studies include both within and between therapist comparisons, they typically have greater power than similar parallel groups cluster randomized trials [82]. A range of effect sizes for LyssnCBT on client outcomes (from 0.05 to 0.25) and therapist sample sizes were examined. Given the proposed design of 5 clinics, 50 providers, 1,875 total clients, and 18,750 repeated measures, the current design has power of 0.80 or more to detect LyssnCBT effects of *d* = 0.15 or greater. This would represent a ‘small’ effect, but given the number of clients who receive CBT services in a given year, this would still entail a large effect in the population over time [83].

## Data management

All data will be recorded using a secure, password protected, HIPAA compliant cloud platform (LyssnCBT). The platform will be hosted on Amazon Web Services (AWS) and require two-factor authentication to ensure the security of all sensitive patient data. Lyssn maintains a Business Associate Agreement (BAA) with AWS to ensure that both parties are adhering to HIPAA guidelines. This platform streams data directly to secure data storage, ensuring that recordings do not reside on less secure therapist computers or tablets. All hard disks utilize full hard drive encryption (in compliance with HIPAA guidelines) and client identifiers have a second level of encryption in the database tables. This cloud-based recording system has already been built and has been used to securely record and store sessions as part of R44 DA046243. Once files have been recorded, they are then processed on the cloud-based platform. This processing includes speech signal processing methods, voice activity detection, speaker segmentation (or diarization), and automated speech recognition (ASR). LyssnCBT therapist competence ratings and speech feature data will then be used in the computer-generated summary reports. These summary reports will be viewable on the same secure, HIPAA compliant cloud platform used for recording. Therapists and supervisors will view the LyssnCBT feedback reports together during supervision, and therapists may also choose to log in to view feedback metrics independently. All other data and source materials are only accessible to the researchers.

The research team will only have access to individually identifiable private information (e.g., name and contact information) on an as-needed basis, such as to confirm that recordings are only being copied for sessions from consented therapists and that LyssnCBT feedback reports are given to the appropriate therapist only.

Data transfer of all files will be protected using strong Advanced Encryption Standard (AES) encryption; electronic data will only be maintained on network servers and computers that incorporate security protections; any hard copies that contain identifiable data will storedin locked file cabinets; subject-identifiable information will be replaced with identification numbers at the earliest possible time; and subject-identifiable information and the link to identification numbers will be kept separately from data.

## Discussion

Artificial intelligence holds great promise for advancing our ability to evaluate therapist skills at scale, providing a view inside the black box of psychotherapy as it is delivered in routine care. This study will employ user-centered design to engage community stakeholders in refining a tool to evaluate and support clinicians’ CBT skills, and then evaluate the impact of that tool on CBT skill and client outcomes in a publicly funded mental health care system. The study will also examine the hypothesized mechanism by which LyssnCBT affects clinician and client outcomes. It will be among the first studies to test artificial-intelligence generated metrics and tools to improve skills and client outcomes in routine mental health care. Outcomes will have a significant impact on the advancement of strategies to implement mental health EBPs at scale and with fidelity, which in turn may have positive impacts for broader accessibility of these treatments. Trial results will be reported in peer-reviewed publications, at scientific presentations, and through open presentations to the community mental health system within which this research will be conducted.

### Potential problems and alternative strategies

It is possible that technology problems (e.g., lack of computer or computer access) will require a more extensive period of system enhancements. The study team has extensive past history working within DBHIDS and similar sites, and the pilot data suggests that sites have the necessary, basic technology infrastructure. However, the investigators will be attentive to these technical issues during the Phase 1 research and have budgeted for just this possibility. Provider turnover and client no-shows could affect the retention rate in the randomized trial and/or increase missing data, reducing statistical power and increasing standard error. Additionally, recruitment for the randomized trial may be slower than expected. The team will proactively employ several strategies to mitigate this risk. If the study flow does not support the recruitment targets by the end of month 9 of Phase 2, the team will work with the recruited agencies and DBHIDS leadership to either identify new providers for recruitment at existing study sites or expand recruitment effort to additional sites.

### Potential for impact

The DBHIDS network includes over 300 agencies. Conservatively assuming that each agency has 10 therapists and each therapist sees 30 clients per week, that is 90,000 sessions per week, approximately 4.5 million per year – in *one* publicly-funded behavioral health system in a single large American city. There are currently no feasible methods for estimating the quality of even a fraction of those sessions. The current research builds upon a robust, existing platform and lays the groundwork for a feasible, technology-enabled assessment of CBT intervention quality at scale, which can inform performance-based feedback for training, supervision, and quality assurance. The combination of the team, the project, and the partnership with DBHIDS presents a unique opportunity to massively scale up quality monitoring of CBT interventions, and the underlying methodologies would lay a foundation for psychosocial intervention quality monitoring and feedback in general. The implications of this research would be improved outcomes for clients, improved support for therapists, and improved quality assurance processes for behavioral healthcare systems.

## Data Availability

Not applicable.

## Abbreviations

AI: Artificial Intelligence
AIM: Acceptability Intervention Measure
BCI: Beck Community Initiative
CBT: Cognitive Behavioral Therapy
CMH: Community Mental Health
CTRS: Cognitive Therapy Rating Scale
DBHIDS: Department of Behavioral Health and Intellectual disAbility Services
EBPs: Evidence-Based Psychotherapies
FIM: Feasibility of Intervention Measure
GAD-7: Generalized Anxiety Disorder 7
IAM: Intervention Appropriateness Measure
ICC: Intraclass Correlation
IT: Information technology
MAR: Missing at Random
MI: Motivational Interviewing
ML: Machine learning
PHQ-9: Patient Health Questionnaire 9
SAU: Services as Usual
SP: Standardized patient
UCD: User-Centered Design
UI: User Interface
